# Attractive toxic sugar bait (ATSB) methods decimate populations of *Anopheles *malaria vectors in arid environments regardless of the local availability of favoured sugar-source blossoms

**DOI:** 10.1186/1475-2875-11-31

**Published:** 2012-02-01

**Authors:** John C Beier, Günter C Müller, Weidong Gu, Kristopher L Arheart, Yosef Schlein

**Affiliations:** 1 Department of Epidemiology and Public Health, University of Miami Miller School of Medicine, Miami, FL 33136, USA; 2 Department of Microbiology and Molecular Genetics, Kuvin Centre for the Study of Infectious and Tropical Diseases, Faculty of Medicine, Hebrew University, Jerusalem, Israel; 3 Division of Infectious Diseases, University of Alabama, Birmingham, AL 35294, USA; 4 Outbreak Surveillance and Analytics Team, Enteric Diseases Epidemiology Branch, Division of Foodborne, Waterborne, and Environmental Diseases, National Center for Emerging and Zoonotic Infectious Diseases, Centers for Disease Control and Prevention, Atlanta, GA 30329, USA

**Keywords:** Sugar feeding, Vectorial capacity, Malaria, Attractive toxic sugar baits (ATSB), Outdoor mosquito control, *Anopheles sergentii*

## Abstract

**Background:**

Attractive toxic sugar bait (ATSB) methods are a new and promising "attract and kill" strategy for mosquito control. Sugar-feeding female and male mosquitoes attracted to ATSB solutions, either sprayed on plants or in bait stations, ingest an incorporated low-risk toxin such as boric acid and are killed. This field study in the arid malaria-free oasis environment of Israel compares how the availability of a primary natural sugar source for *Anopheles sergentii *mosquitoes: flowering *Acacia raddiana *trees, affects the efficacy of ATSB methods for mosquito control.

**Methods:**

A 47-day field trial was conducted to compare impacts of a single application of ATSB treatment on mosquito densities and age structure in isolated uninhabited sugar-rich and sugar-poor oases relative to an untreated sugar-rich oasis that served as a control.

**Results:**

ATSB spraying on patches of non-flowering vegetation around freshwater springs reduced densities of female *An. sergentii *by 95.2% in the sugar-rich oasis and 98.6% in the sugar-poor oasis; males in both oases were practically eliminated. It reduced daily survival rates of female *An. sergentii *from 0.77 to 0.35 in the sugar-poor oasis and from 0.85 to 0.51 in the sugar-rich oasis. ATSB treatment reduced the proportion of older more epidemiologically dangerous mosquitoes (three or more gonotrophic cycles) by 100% and 96.7%, respectively, in the sugar-poor and sugar-rich oases. Overall, malaria vectorial capacity was reduced from 11.2 to 0.0 in the sugar-poor oasis and from 79.0 to 0.03 in the sugar-rich oasis. Reduction in vector capacity to negligible levels days after ATSB application in the sugar-poor oasis, but not until after 2 weeks in the sugar-rich oasis, show that natural sugar sources compete with the applied ATSB solutions.

**Conclusion:**

While readily available natural sugar sources delay ATSB impact, they do not affect overall outcomes because the high frequency of sugar feeding by mosquitoes has an accumulating effect on the probability they will be attracted to and killed by ATSB methods. Operationally, ATSB methods for malaria vector control are highly effective in arid environments regardless of competitive, highly attractive natural sugar sources in their outdoor environment.

## Background

Attractive toxic sugar bait (ATSB) methods are a new form of vector control that kill female and male mosquitoes questing for essential sugar sources in the outdoor environment [1-7]. ATSB solutions consist of fruit or flower scent as an attractant, sugar solution as a feeding stimulant, and oral toxin to kill the mosquitoes. ATSB solutions that are sprayed on small spots of vegetation or suspended in simple removable bait stations attract mosquitoes from a large area and the mosquitoes ingesting the toxic solutions are killed. The ATSB methods developed and field-tested in Israel demonstrate how they literally decimate local populations of different anopheline and culicine mosquito species [1-5]. Similar successful ATSB field trials have also controlled *Culex quinquefasciatus *from storm drains in Florida, USA [6] and *Anopheles gambiae *s.l. malaria vectors in Mali, West Africa [7]. The new ATSB methods are highly effective, technologically simple, low-cost, and circumvent traditional problems associated with the indiscriminate effects of contact insecticides [8] by narrowing the specificity of attraction to sugar-seeking mosquitoes and by using environmentally safe oral toxins such as boric acid, that is considered to be only slightly more toxic to humans and other vertebrates than table salt [9].

ATSB methods work by competing with available natural plant sugar sources, which are an essential source of energy for females and the only food source for male mosquitoes [10,11]. Mosquitoes are highly selective in their attraction to locally available flowering plants and other sources of sugar including fruits, seedpods, and honeydew [12-14] and the availability of favourable natural sugar sources strongly affects mosquito survival [13]. All of the above-noted ATSB field trials used juices made from local natural fruits to successfully divert sugar-seeking mosquitoes from their natural sources of plant sugars. Between 50 and 90% of the local female and male mosquitoes feed on ATSB solutions within the first few days after applications, as inferred from data at control sites where the same attractive bait solutions are applied without toxin but containing coloured food dye markers, which are readily apparent in sugar-fed mosquitoes [1].

The present study is on *Anopheles sergentii*, the most common and abundant *Anopheles *species in Israel and the main vector of malaria in the Afro-Arabian zone [15,16]. This mosquito species was the main vector responsible for malaria outbreaks [17-19] before the elimination of malaria parasite transmission from Israel in the 1960s [20-22].

The objective of this study was to determine the relationship between the efficacy of ATSB control and the availability of natural plant sugar sources. As demonstrated in a recent comparative study of *An. sergentii *in sugar-rich and sugar-poor oases in Israel, the availability of natural plant sugar sources affects mosquito fitness, population dynamics, and malaria vector capacity [23]. Accordingly, a single application of ATSB was made in the same two relatively small, isolated, and uninhabited sugar-rich and sugar-poor oases. Another larger oasis with high densities of *An. sergentii *was not sprayed and served as a control site for the ATSB field trial.

## Methods

### Study sites

The study was conducted at three oases located within the depression of the African-Syrian Rift Valley, in the northern part of the Arava Valley, about 25 km south of the Dead Sea. The shoreline of the Dead Sea is about 400 m below sea level while the central Arava Valley rises to about 200 m above sea level before it is again descending towards the Red Sea. The region belongs to the Sahara-Arabian phyto-geographical zone [24]. The area is an extreme desert with occasional natural oases centred on springs and artificial agricultural oases created by irrigation; the conditions in these sites are tropical [25]. The climate is arid with an average humidity of 57% and annual winter rains averaging of 50-100 mm. The average temperature ranges from 20°C from the end of September to early April and to 30°C from May to August [26,27]. The area is known for its rich mosquito fauna dominated by *An. sergentii, Ochlerotatus caspius *and *Culex pipiens *[28].

Field experiments were conducted at three oases. Two of the oases included small, unnamed and uninhabited oases, 5 km apart in the Arava Valley. As recently described [23], the environments of the two oases are very similar except for the availability of sugar sources. In one of the oases (termed "sugar-rich oasis"), there were two flowering *Acacia raddiana *trees that were the preferable source of sugar for the mosquitoes [14]. In contrast, there were no flowering plant blossoms in the other oasis (termed "sugar-poor oasis"). Both sites covered areas of about 5 ha, included small fresh-water springs surrounded by dense non-flowering vegetation which was largely grazed out by camels and donkeys with no visible plant sugar sources during the period of field experiments [23,24]. Neot Hakikar oasis served as an untreated control site. It is located about 20 km north of the small oases and is the largest natural oasis in southern Israel and the Dead Sea region. In the eastern more agricultural part of the oasis a small settlement is located with gardens, vast fields and greenhouses. The western, much more natural part is a nature reserve with a mixture of salt marshes, wet and dry Salinas (ie areas high in salt content with specialized plant communities) and fresh-water springs surrounded by riparian vegetation largely dominated by *Phragmites australis *L. Gramineae and *Carex *sp. L. Cyperaceae. This natural vegetation, crossed by a drainage canal, is partially overgrown by reeds and sedges. On the dry banks of the canal vegetation is dominated by groves and thickets of trees and bushes like *Tamarix nilotica *and *Tamarix passerinoides *(Tamaricaceae), *Prosopis farcta *(Mimosaceae), *Nitraria retusa *(Nitrariaceae) and chenopod bushes like *Atriplex halimus, Atriplex leucoclada, Suaeda asphaltica, Suaeda fruticosa *(Chenopodiaceae). At the time of the experiment some *T. nilotica *and *P. farcta *bushes were flowering.

### Preparation of ATSB solutions

The ATSB bait solution used in the sugar-rich and sugar-poor oases consisted of ~75% juice of over-ripe to rotting prickly pear cactus (*Opuntia ficus-indica*, Cactaceae), 5% V/V wine, 20% W/V brown sugar, 1% (W/V) BaitStab™ concentrate (a product containing antifungal and antibacterial additives produced by Westham Innovations LTD, Tel Aviv, Israel) and boric acid 1% (W/V) [29]. The solution was ripened outdoors for 48 h in covered buckets before adding the BaitStab™ and the boric acid. In this study, prickly pear cactus fruit (*Opuntia ficus-indica*) was used because it was locally abundant and known to be highly attractive for both sand flies [29] and mosquitoes (Schlein and Muller, unpublished).

### Field application of ATSB solutions

The ATSB solution was sprayed with a 16-l back-pack sprayer (Killaspray, Model 4526, Hozelock, Birmingham UK) in aliquots of ~80 ml on 1 m^2 ^spots at distances of ~3 m on the vegetation surrounding the fresh-water springs of the two isolated oasis (sugar-rich and sugar-poor). Predominant types of non-flowering plants sprayed at the two sites were *P. australis, Atriplex *sp. and *Suaeda *sp. As a strategy to minimize potential harm to non-target insects, the predominant natural sugar source for *An. sergentii*, the flowering *A. raddiana *trees, present in the sugar-rich oasis were not sprayed. One sprayer completed the applications in less than 1 h per site. No bait solution was sprayed at the control site Neot Hakikar.

### Study design and methods for the ATSB field trial

The field trial was conducted over a period of 47 days, from 1 November to 17 December, 2009. During this period, at each of the three study sites, adult mosquitoes were sampled at two-day intervals (a total of 24 times) using six CDC UV traps (Model 1212, John W. Hock, Gainesville, FL) without attractants in fixed positions surrounding the available fresh-water springs. ATSB bait solutions were sprayed on day 12 of the field experiment. Collected mosquitoes were sexed, identified to species, and the physiological age of female mosquitoes was determined by dissecting ovaries and counting the number of dilatations [30].

### Statistical analysis

To evaluate impacts of ATSB on mosquito populations, captures of *An. sergentii *were examined at four intervals (1-12, 13-24, 25-36, and 37-47 days). A logistic regression was used to examine the proportion of females with three or more gonotrophic cycles in each oasis over time. Contrasts were used to test for significant changes from the pre-treatment period in each oasis. Separate Poisson regressions were used to analyse the number of male and female *An. sergentii *caught in the light traps over time in the three oases. Contrasts were used to compare the control oasis with the poor and rich oases at each time.

### Estimation of vectorial capacity

Vectorial capacity (VC). defined as the average number of infectious bites the mosquito could potentially deliver over her lifetime, was used to estimate the impact of ATSB on the potential for malaria parasite transmission:

$$VC=\frac{mp^{EIP}}{-T^{2}\log\left(p\right)}$$

Where *m *was the number of mosquitoes per person, *T *was the estimated duration of the gonotrophic cycle [23]. *EIP *was the extrinsic incubation period of malaria parasites in mosquitoes assuming to be 10 days [31]. *p *was the survival rate estimated based on parous rates *r*.

$$p=\sqrt[{T}]{r}$$

Following Dye [32], VC was compared before and after the intervention. Therefore, only *m *and *p *were separately estimated for the two periods. *m *was estimated as the average number of female mosquitoes caught per trap night.

## Results

**Figure 1:**
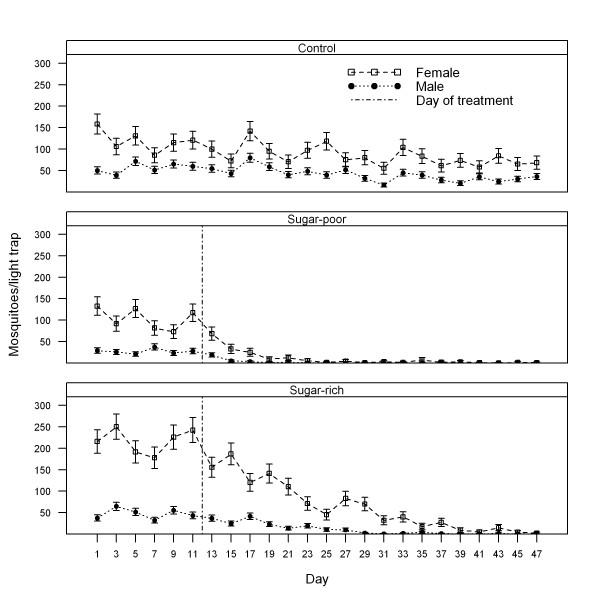
**Averages (± 1 standard error) of light trap captures of female and male *Anopheles sergentii *in three oases (sugar-rich, sugar-poor, and control) from 1 November to 17 December, 2009, in Israel (vertical dot lines in panels indicate the date of implementation of ATSB)**.

At both the sugar-poor and sugar-rich oases, a single application of ATSB on day 12 reduced densities of female *An. sergentii *by over 95% and practically eliminated male *An. sergentii *(Figure 1). Densities of female and male *An. sergentii *in the sugar-poor oasis were immediately reduced by ATSB treatment compared with the more gradual decreases observed in the sugar-rich oasis.

Densities of female *An. sergentii *in the sugar-poor and sugar-rich sites from the pre-treatment period (days 1-12) to the post-treatment period (days 13-47) decreased over 75-fold and 20-fold, respectively, compared to less than a two-fold natural decrease at the control site that did not receive ATSB treatment. At the control site, densities of female *An. sergentii *averaged 119.42 ± 9.98 before day 12 and 83.52 ± 5.30 from days 13-47. At the sugar-poor oasis, densities of female *An. sergentii *averaged 103.81 ± 10.20 before ATSB treatment and 9.97 ± 4.02 post-treatment. At the sugar-rich oasis, densities of female *An. sergentii *averaged 217.19 ± 11.54 before ATSB treatment and 63.07 ± 13.63 post-treatment. For all but two comparisons of the control oasis with either rich or poor oases the differences were significant at *p *< 0.001 for females after the treatment was applied: control was significantly higher than poor and lower than rich.

Similarly, for male *An. sergentii*, densities decreased about 15-fold and four-fold from the pre-treatment to the post-treatment period in the sugar-poor and sugar-rich sites, respectively, compared to only a one-fold decrease at the control site. At the control site, densities of male *An. sergentii *averaged 56.22 ± 4.77 before day 12 and 40.18 ± 3.59 from days 13-47. At the sugar-poor oasis, densities of male *An. sergentii *averaged 27.36 ± 2.37 before ATSB treatment and 1.75 ± 1.05 from post-treatment. At the sugar-rich oasis, densities of male *An. sergentii *averaged 47.42 ± 4.88 before ATSB treatment and 10.64 ± 3.10 post-treatment. After treatment, males were significantly lower in both poor and rich oases compared with the control oasis at *p *< 0.001.

Table 1 shows, according to pre-treatment days 1-12 and the three post-treatment periods, how ATSB treatment in the sugar-poor and sugar-rich oases affected the proportion of females classified according to gonotrophic cycles (0, 1, 2, 3, and > 3). ATSB treatment reduced the proportion of older more epidemiologically dangerous mosquitoes (three or more gonotrophic cycles) by 100% and 94.9%, respectively, in the sugar-poor and sugar-rich oasis. In the control group the proportion of females with three or more gonotropic cycles increased slightly but not significantly over time. At the sugar-poor site, the proportion of females with three or more gonotropic cycles was significantly reduced compared to pre-treatment levels at 13-24 days (*p *= 0.011), at 25-35 days (*p *= 0.014), and at 36-47 days (*p *< 0.001). At the sugar-rich site, the number of females with three or more gonotropic cycles was significantly reduced in the first week post-treatment (*p *= 0.001) and at the subsequent measurement times (*p *< 0.001 for both times).

**Table 1:** Age structure, population parameters, and vectorial capacity (VC) of female *Anopheles sergentii *before and after ATSB treatment on day 12

Site	Intervals of days	Total dissected	Number of gonotrophic cycles (%)					Density/day	Parous rate	Survival rate	VC
			0	1	2	3	> 3				
Control	1-12	180	30	23	14	11	22	119.42	0.70	0.84	33.99
	13-24	180	36	19	12	9	24	96.08	0.64	0.80	16.85
	25-36	180	29	18	12	10	30	85.97	0.71	0.84	25.69
	37-47	180	24	17	15	12	32	68.50	0.76	0.87	32.00
											
Sugar poor	1-12	180	41	32	14	6	7	103.81	0.59	0.77	11.19
	13-24	180	73	18	5	2	1	25.42	0.27	0.52	0.08
	25-36	141	81	16	2	1	0	3.14	0.19	0.44	0.00
	37-47	92	88	11	1	0	0	1.36	0.12	0.35	0.00
											
Sugar rich	1-12	180	27	19	14	9	30	217.19	0.73	0.85	79.00
	13-24	180	39	20	17	8	15	130.83	0.61	0.78	16.33
	25-36	180	67	22	7	3	2	47.89	0.33	0.58	0.37
	37-47	171	74	18	6	1	1	10.50	0.26	0.51	0.03

Table 1 also shows how ATSB treatment markedly reduced female *An. sergentii *densities, parous rates, survival rates and vectorial capacity. Compared with the control site, while female *An. sergentii *densities decreased less than two-fold as indicated above, parous rates, survival rates, and vectorial capacity remained fairly constant throughout the monitoring period. From the pre-treatment period (days 1-12) to the last period of post-treatment monitoring (days 37-47), the parous rates decreased from 0.59 to 0.12 at the sugar-poor site and decreased from 0.73 to 0.26 at the sugar-rich site. During the same periods, the survival rates decreased from 0.77 to 0.35 at the sugar-poor site and decreased from 0.85 to 0.51 at the sugar-rich site. Malaria vectorial capacity was reduced from a pre-treatment level of 11.2 to 0.0 (last two monitoring periods, days 25-35 and days 37-47) at the sugar-poor oasis and from a pre-treatment level of 79.0 to 0.03 (last monitoring period, days 37-47) at the sugar-rich oasis. Reduction in VC to negligible levels was observed days after ATSB application in the sugar-poor oasis but not until after 2 weeks in the sugar-rich oasis.

## Discussion

This field trial shows that a single application of ATSB solution by plant spraying at the two oases treatment sites markedly reduced the relative abundance of *An. sergentii *populations and their longevity. Densities of adult females and males, and the proportion of "older" more dangerous females were reduced by 95% or more. Not unexpectedly, the impact of the ATSB treatment is comparable to that demonstrated in previous field trials [1-7].

The comparison of ATSB spraying of non-flowering vegetation in the sugar-rich and sugar-poor oases allowed experimental testing of the hypothesis that natural sugar resources compete with the ATSB. As expected, ATSB application in the sugar-poor oasis reduced densities of female *An. sergentii *by 95% within 2 weeks. In contrast, it took 4 weeks in the sugar-rich oasis for ATSB application to reduce densities of female *An. sergentii *by 95%. The difference of 2 weeks to 95% population reduction in the sugar-rich oasis, likely due to a reduced frequency of mosquito exposure to ATSB, represented competition with attractive natural sources.

The finding that, regardless of the available natural sugar resources, ATSB use can substantially reduce mosquito densities in arid environments is likely due to high frequencies of mosquito sugar-feeding [33,34]. Most female and male mosquitoes likely encounter sprayed ATSB solutions and feed at least once during their lifespan (Figure 1). When ATSB solutions are sprayed on non-flowering vegetation as a strategy to reduce overall impact on non-target insects [7], the sprayed areas largely represent favourable outdoor mosquito resting microenvironments and not sugar-feeding centres containing attractive flowering plants. The probability that mosquitoes encounter and feed on sprayed ATSB solution at their outdoor resting microhabitats is high because these are specific locations where mosquitoes spend most of their time.

This study demonstrates for the first time under experimental field conditions how a single application of ATSB can reduce malaria VC from relatively high to negligible levels. Based on ATSB field trials to date [1-7], it is likely that this new approach can also be used in different malaria endemic environments to impact entomological inoculation rates (EIRs) and epidemiological parameters of malaria in humans. Remaining are challenges in the areas of: 1) product development, to standardize attractive baits; 2) deployment methods, to determine the seasonal timing and coverage needed to maximize efficacy while minimizing potential costs and any potential harm to non-target invertebrates; and 3) controlled field trials, to determine how ATSB strategies can be used in combination with existing vector control methods to additionally impact EIRs, especially in eco-epidemiological situations where the continuing problems of malaria cannot be solved using current vector control methods.

## Conclusions

This study provides further evidence that ATSB methods can effectively target and kill sugar-feeding anopheline mosquitoes, and shows how available natural sugar resources used by mosquitoes in arid environments compete with applied ATSB solutions. While abundant sugar resources in the sugar-rich oasis delayed full impacts of ATSB by about 2 weeks, mosquito population reductions of over 95% were none-the-less achieved by a single ATSB application. As well, this study shows for the first time how ATSB can reduce malaria VC from relatively high to negligible levels, with only minimal differences due to sugar-poor and sugar-rich environmental conditions. Overall, this demonstration of how even single applications of ATSB solutions can operationally decimate populations of anopheline mosquitoes and drive their potential for malaria transmission to near zero levels highlights the importance of ATSB as a promising new tool for outdoor vector control.

## Competing interests

The authors declare that they have no competing interests.

## Authors' contributions

GCM, JCB, WG, and YS conceived and planned the study, interpreted results, and wrote the paper. GCM directed and performed the field experiments and managed the data. KLA and WG analysed the data. All authors read and approved the final manuscript.
